# Physical Fitness of Chinese Primary School Students across the Coronavirus (COVID-19) Outbreak: A Retrospective Repeated Cross-Sectional Study

**DOI:** 10.3390/ijerph19137870

**Published:** 2022-06-27

**Authors:** Wei-Ning Hu, Dong-Yue Li, Wing-Kai Lam, Yi Wang, Duo Wai-Chi Wong, James Chung-Wai Cheung

**Affiliations:** 1 Physical Education Group, Yuxian School, Guangzhou 511400, China; weinghu@163.com; 2 Department of Physical Education, Guangzhou University, Guangzhou 510006, China; lidongyue@gzhu.edu.cn; 3 Sports Information and External Affairs Centre, Hong Kong Sports Institute, Hong Kong 999077, China; gilbert.lam@connect.polyu.hk; 4 Department of Physical Education, Renmin University of China, Beijing 100872, China; 5 Sports and Social Development Research Center, Renmin University of China, Beijing 100872, China; 6 Department of Biomedical Engineering, Faculty of Engineering, The Hong Kong Polytechnic University, Hong Kong 999077, China; james.chungwai.cheung@polyu.edu.hk; 7 Research Institute for Sports Science and Technology, The Hong Kong Polytechnic University, Hong Kong 999077, China

**Keywords:** 2019-nCoV, physical activity, physical education, middle childhood, social distancing, muscular strength, flexibility

## Abstract

Social distancing measures against COVID-19 imposed restrictions on students that may have affected their physical health and fitness. The objective of this study was to investigate the change in physical fitness of primary school students across the coronavirus outbreaks from 2019 to 2021. This was a retrospective repeated cross-sectional study. We obtained the annual physical and fitness assessment data measured every November for all students at the same primary school in Guangzhou, China. There was a total of 6371 observations in the dataset for three years. The physical fitness of the students was evaluated with an overall physical fitness score, body mass index (BMI), lung vital capacity, physical flexibility (via a sit-and-reach test) and sports task performances (sprint, shuttle run, rope-jumping, and sit-up). Generalised estimating equations were used to determine any significant changes from 2019 to 2021, adjusted for confounders. After the COVID-19 outbreak in 2021, there was a significant elevation in BMI of 0.64 kg/m^2^ in 2020 and 0.39 kg/m^2^ in 2021 (*p* < 0.001). The overall physical fitness score was significantly increased by 2.1 and 4.1 points, respectively, in 2020 and 2021 (*p* < 0.001). Lung vital capacity and rope-jumping performance were significantly improved in both 2020 and 2021 compared with 2019, and sit-up performance was marginally significantly improved in 2020 and significantly improved in 2021. However, students demonstrated poorer flexibility and sprint and shuttle run performance in 2021 compared with 2019. A health promotion programme during and after COVID-19, including online physical education classes, television broadcasts, and a rope-jumping campaign, could account for these positive outcomes, along with the ease of administering rope-jumping and sit-ups at home.

## 1. Introduction

Coronavirus disease 2019 (COVID-19 or 2019-nCoV) represents the greatest global threat in human history after World War II. The highly contagious and infectious virus was first reported in Wuhan, Hubei Province of China, in December 2019 and was declared a global pandemic by the World Health Organization in March 2020. As of 8 February 2022, there have been 390 million confirmed cases of COVID-19, resulting in 5.7 million deaths worldwide [1]. The average fatality rate was estimated to be 4% but could be as high as 20% in some countries/regions [2,3]. 

Social (or physical) distancing is one of the cost-effective policy measures for epidemic mitigation/spread [4] and has been implemented at different levels. More than 130 countries or regions imposed border closures or restrictions that have successfully helped block the spread of coronavirus around the world [5]. These measures included travel restrictions, screening, quarantine/isolation—a circuit-breaker for flights—and 90% of commercial flights were grounded [6]. On 23 January 2020, Wuhan province announced a ban on flights and shut down the city for 76 days. From epidemic to pandemic, various social distancing measures were put in place across countries and cities. In addition to the suspension of intra-city public transport in Mainland China, the government also closed the entertainment/sport premises, banned gatherings, limited dining time, and encouraged special work arrangements (work from home) to minimize outdoor mobility and the transmission of infection. 

Nevertheless, various social distancing measures have hampered the time and freedom for physical activities [7,8]. For example, sports facilities, including gyms, fitness clubs, and public parks, were forced to close, while work from home arrangements and the mask mandates [9] discouraged people from exercising or walking outdoors. As a result, people increased their sitting time by 28% [10] and perceived that their fitness had declined remarkably by half [11]. In the United States, it was reported that the coronavirus reduced physical activity time by 18.2% [12], compared to 30% in Japan, with a further decline of 15% one year after the outbreak. 

Students were not spared from social distancing measures and were kept out of school/campus. In January 2020, the Ministry of Education of China announced the shutdown of all schools starting in March, including kindergartens, primary, secondary, tertiary (university), and vocational colleges, after the prolonged Chinese New Year holidays and winter break. Although the Zoom generation (Gen Z) [13] has adapted to the virtual classroom and online learning, they experienced social and affective challenges of isolation [14], in addition to the challenges of physical education (PE) and thus exercise time [15]. Physically active students reported a reduction in regular and planned physical exercise by 41.7% during the pandemic, resulting in a decline in physical condition of 38.2% [16]. It was even more problematic for physically inactive and moderately active students [17]. The time for physical exercise might shift into sedentary and screen time [18,19], which is recognised as a cause of de-training in students [20]. The association between physical exercise time and physical fitness is indubitable. 

The objective of this study was to investigate the influence of coronavirus (COVID-19) on the physical fitness of primary school students that could be used to inform educators and policy-makers. We hypothesised that the physical fitness of the students would decline after the outbreak and shutdown arrangements and could be recovered in the aftermath [21]. 

## 2. Materials and Methods

### 2.1. Participants

This was a retrospective study approved by the Institutional Review Board (Ref. No.: HSEARS20220418001). The study was a repeated cross-sectional design based on physical fitness data for students for three consecutive academic years (2019–2021) collected from one selected primary school in Guangzhou, Guangdong Province, China. Physical fitness assessment with a standard protocol (i.e., the National Student Physical Fitness Standard of China, version 2014) [22] is a mandatory exercise for all primary schools that is conducted every year in China. All students are required to attend the physical fitness assessment in each academic year unless they have applied for an exemption with reasons. 

The physical fitness assessment is carried out during the first to third week of November every academic year by a PE teacher. All students attended the same PE curriculum taught by the same teacher and teaching assistants for the same amount of time. The same PE teacher and trained assistants arranged and conducted the assessments in the same venue on the school campus during regular school hours. Approximately 100 students were fully assessed in one morning/afternoon section. All students wore the same type of school sports uniform and footwear. The standard physical fitness protocol (version 2014) [22] included body mass index (BMI) measurement, lung vital capacity measurement, 50 m sprint test, sit-and-reach flexibility test, and 1 min rope-jumping test. One minute sit-ups were performed by grade 3 to 6 students only. Additionally, only grade 5 to 6 students were required to perform a 50 m × 8 shuttle run test. The number of assessments for each grade is listed in Table 1.

### 2.2. Assessment Indicators and Tasks

#### 2.2.1. Overall Physical Fitness Score

According to the National Student Physical Fitness Standard of China (version 2014) [22], the overall physical fitness score is calculated by the weighted sum of five to seven normalised indicators depending on the grade, as shown in Table 1. The maximum overall physical fitness score was 100 points plus 20 bonus points where applicable. The normalization tables for the indicators are listed in the Supplementary Material (Table S1). 

#### 2.2.2. BMI

Body mass and body height were measured by an ultrasound measuring instrument and scale (sH-200, Zhengzhou Shanghe Electronic Technology Company Limited, Zhengzhou, China) with a body height and mass precision of 0.01 cm and 0.1 kg, respectively. The students stood barefoot on the scale in an upright position, looked straight ahead, and had their heels together during the measurement. The heel, sacrum, and the midpoint between shoulder blades were joined in a vertical line. The height and mass measurements were rounded to the nearest 0.1 cm and 0.1 kg, respectively. BMI was calculated by dividing the mass (in kg) by the square of the height (in m).

#### 2.2.3. Lung Vital Capacity

The lung vital capacity is the maximum expelled air volume after a maximum inhalation. It was measured by an electronic spirometer (wqs-8888, Shanghai Wanqing Electronic Company Limited, Shanghai, China) with a capacity of 10 L and resolution of 1 mL. During the measurement, the student stood upright, with his/her head slightly tilted backwards. Next, the student performed a maximum inhalation and exhaled slowly into the mouthpiece until no more air came out. The measurement was repeated twice, and the best performance was recorded. This protocol demonstrated sufficient acceptability and reproducibility in a previous study of preschool children [23].

#### 2.2.4. Fifty Meter Sprint

The sprint test was conducted after three PE classes during the semesters when the school was not in lockdown (weekdays of the first three weeks of November). The students performed different warm-up activities, such as stretching and then jogging approximately 400 m to prepare for the tasks of the fitness test. Before the 50 m sprint test, the students warmed up for about three minutes and were readied in a standing start position. The students ran on a straight running track for 50 m as fast as possible toward the finish line. The time for the run was recorded by a stopwatch (pc80, Shenzhen Timestar Electronic Co. Ltd., Shenzhen, China) to the nearest 0.1 s.

#### 2.2.5. Sit-and-Reach Flexibility Test

The sit-and-reach test was measured by an electronic box tester (HWD21-1231, Li-Ning (China) Company Limited, Beijing, China) with a range of 60 cm and a resolution of 0.1 cm. Before the test, the students sat barefoot on the ground, fully extended their knees, heels together, and stepped on the pedal of the tester box. Their feet were placed approximately 10 to 15 cm apart. The students slowly leaned their trunk forward and extended their arms during the test. They were asked to reach forward and slowly push a cursor with their middle fingers for as far as possible until the cursor could not be moved any further forward. The students were not allowed to make an impulsive move for extra distance. The measurement was repeated twice, and the best performance was recorded.

#### 2.2.6. One Minute Rope-Jumping

The test was conducted and measured by a counter jumping rope (Li-Ning (China) Company Limited, Beijing, China). The baseline rope length was 2.8 m. The length was adjusted by raising the handle to chest level in a preparatory posture when stepping on the rope. The rope was tossed by the students themselves at their self-selected speed. The students were asked to jump continuously and as much as he/she could in one minute in one trial. Within the given one minute, they were allowed to resume and continue the rope-jumping task if they paused or tripped. However, the “tripped jump” was not counted. The total number of jumps in one minute was recorded by the counter.

#### 2.2.7. One Minute Sit-Ups

Before the test, the students assumed a supine position on a flat surface with a floor mat. Their knees were bent at 90° and slightly apart while their fingers touched the ears. Another classmate helped secure the lower limb of the participating student at the ankle joint. The sit-up manoeuvre involved the elevation of the trunk until the elbows touched the knees before a return to the starting position. The students were asked to perform as many repetitions as possible for one minute during the test. An examiner counted the number of repetitions, which was recognised when the elbows touched or exceeded the level of the knees and the shoulder blades returned to the ground. The trial was only conducted once. The one minute sit-up test was conducted by grade 3 to 6 students.

#### 2.2.8. Fifty Meter × 8 Shuttle Run

The test was conducted on a sports ground with straight running tracks. The width of the track was 1.22 m, while the distance between the start and finish lines was 50 m. Poles (1.2 m tall) were set at the start and finish lines. Before the run, the students performed warm-ups by stretching and jogging. Next, the students were readied in a standing start position. They were then asked to run as fast as possible toward the finish line, turn around the pole, return to the starting line, then repeat the process three times (i.e., 50 m × 8 shuttles). The time was recorded when the chest crossed the line and rounded to the nearest 0.1 s. The trial was conducted once. The test was conducted by grade 5 to 6 students.

### 2.3. Statistical Analysis

Before the analysis, the basic information of the students was compiled, including the number of students, gender, age, body height and mass, and BMI, for each of the assessment years. 

The primary dependent variables in the analysis were the overall physical fitness score, BMI, and lung vital capacity (in mL), and the secondary variables were the performance measures, including the 50 m sprint (s), one minute rope-jumping (jumping count), sit and reach test (cm), one minute sit-ups (repetitions), and shuttle run (s). 

To investigate whether there were significant differences in the dependent variables across the COVID-19 outbreak (i.e., the academic years of 2019, 2020, and 2021), generalised estimating equations (GEE) with an unstructured correlation matrix were used. The outcome responses were assumed to have a Gaussian distribution, except that of rope-jumping and sit-ups tasks (negative binomial with log link). Covariates or confounding factors included age, gender, and BMI. The repeated cross-sectional study involved students advancing through the grades, freshmen, and students leaving the study because of graduation at each timepoint (academic year). The GEE is a marginal model that can accommodate the nesting of repeated observations by providing a robust standard error estimation towards the average effect of the predictors. 

**Figure 1:**
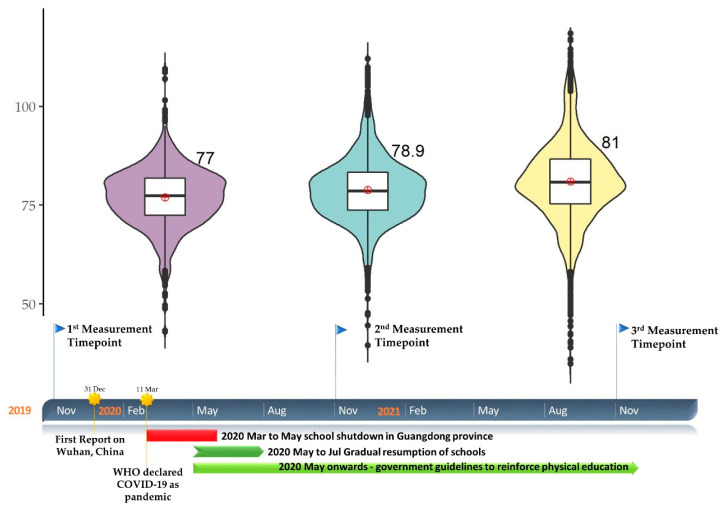
The overall physical fitness scores of primary students in 2019, 2020, and 2021 along with the timeline of COVID-19 events, social distancing policy, and education arrangements.

**Figure 2:**
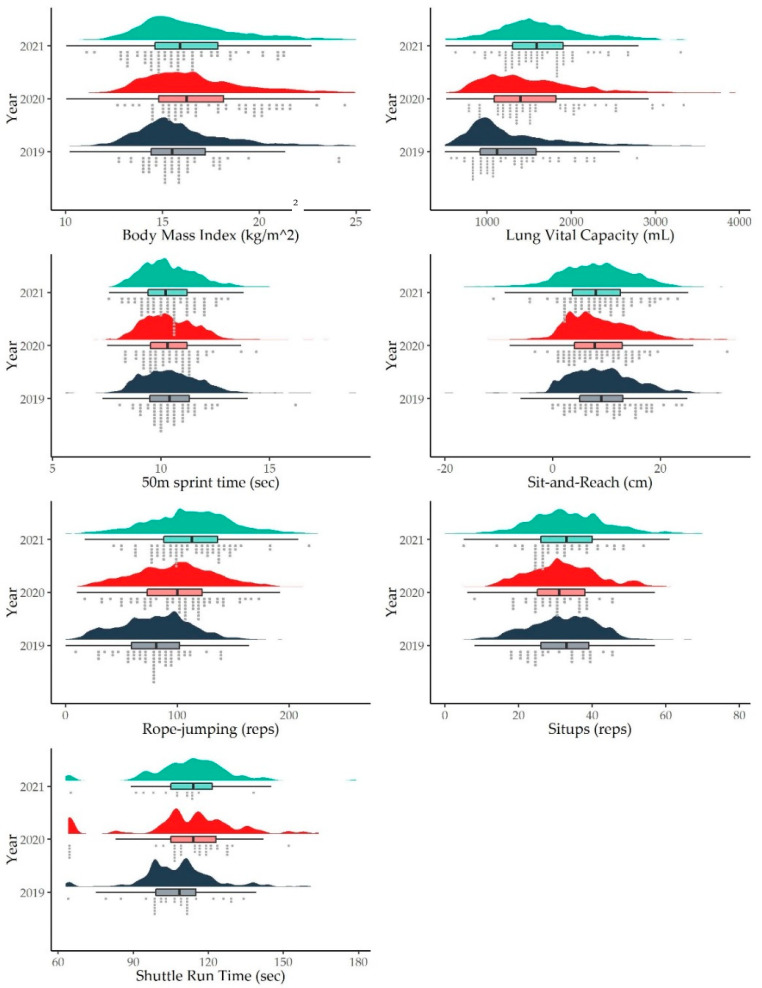
Outcome measures and their distribution for the different years of measurement (purple, 2019; red, 2020; cyan, 2021).

Statistical analysis was conducted using the SPSS package (ver. 26, IBM, New York, NY, USA). The significance level (α) was set as *p* = 0.05. The overall physical fitness score was presented with the timeline in a violin plot (Figure 1), whereas the other results were visualised using raincloud plots (Figure 2). 

## 3. Results

### 3.1. Basic Information

As shown in Table 2, the three assessments conducted in 2019, 2020, and 2021 had a total of 6371 observations. Ten observations of the data were removed due to missing values. The number of exemptions in 2019, 2020, and 2021 were seven (all with medical reasons), nine (seven with medical reasons, two quit/transfer school), and ten (seven with medical reasons, three quit/transfer school), respectively. The number of students in a cohort may not be consistent from year to year due to exemptions, missing data, and new students transferring from other schools.

The average age ranged from 8.7 to 9.1. The ratio of males to females was 1.2. In addition, the BMI in our samples was similar to the 50th percentile for Chinese boys and girls at age 9 (16.2 and 15.7, respectively) [24]. 

### 3.2. Primary Outcome (Overall Score, BMI, Vital Capacity)

The average overall physical fitness score was 77 points in 2019, as shown in Figure 1 and Table 3. There was a significant increase of approximately two points per year (*p* < 0.001) adjusted for other confounders, and the scores seemed to be more dispersed in 2021. Although the BMI demonstrated a significant increase adjusted for other confounders (*p* < 0.001), the magnitude of the change was small and less than 1 kg/m^2^. Moreover, compared to 2019, lung vital capacity values in 2020 and 2021 were significantly higher by 153 mL (95%CI, 130 to 177) and 240 mL (95%CI, 215 to 264), respectively.

Gender, age and BMI were significant confounders for the primary variables. Females had a significantly lower BMI (*p* < 0.001) and lung vital capacity (*p* < 0.001), and an increase in BMI and lung vital capacity was associated with increasing age. Every one unit increase in BMI contributed to a 22.6 mL increase in lung vital capacity adjusted for other factors. 

### 3.3. Secondary Outcome (Sports Task Performance)

As shown in Table 3, the performance scores for rope-jumping and sit-ups improved over the years. Compared to 2019, students achieved 1.23 and 1.39 more jumps in 2020 and 2021, respectively, and the differences were significant (*p* < 0.001) after adjusting for confounders. In addition, the students could perform 1.07 more sit-up reps in 2021 compared to 2019 (*p* < 0.001).

Sprint, sit-and-reach, and shuttle run performances deteriorated. In 2021, students required an extra 5.3 s to complete the shuttle run task compared to 2019 (*p* = 0.001). Although times for the 50 m sprint also increased significantly (*p* = 0.001), the effect of the increase was just 0.10 s in 2021. Flexibility, as measured by the sit-and-reach test, also declined significantly by 0.64 cm (95%CI, 0.35 to 0.94) in 2020 and 1.32 cm (95%CI, 0.98 to 1.66) in 2021 after adjusting for confounders.

Gender, age, and BMI imposed significant effects on sprint performance and the sit-and-reach distance. Females demonstrated significantly better flexibility in the sit-and-reach test but required more time to complete the sprint task. It seems that BMI was not associated with rope-jumping and sit-up performance, and age was not a significant confounding factor for shuttle run performance. 

## 4. Discussion

Amidst the disaster brought about by the COVID-19 pandemic, physical inactivity has also been recognised as a pandemic, and is fourth leading risk for mortality [25]. Social distancing measures have induced physical inactivity and sedentary behaviours that could cause the next potential wave of physical health issues in the post-COVID-19 era [26,27]. Physical activity not only plays a vital role in maintaining physical and mental health, but it also helps fight against the coronavirus [8,28]. Physical training is recognised as an effective strategy for mitigating infection and is strongly recommended for those who have recovered from the infection [29]. It can significantly enhance the immune system, reduce the risks of severe and acute syndromes, and counteract co-morbidities [29,30,31]. 

We hypothesised that physical fitness would deteriorate after the shutdown during COVID-19. Since some of our findings did not align with our original hypothesis, we decided to perform a post-analysis of the timeline and health promotion programme. The timeline for critical events during COVID-19, policies, measurement time points and education campaigns is illustrated in Figure 1 aligned with the primary outcome of overall physical fitness score. Our first measurement dataset was collected in November 2019, which was before the outbreak of COVID-19. The novel coronavirus was reported in the Wuhan Province, China on December 2019 and was declared as a pandemic by World Health Organization (WHO) in March 2020. In March 2020, the Guangdong Province announced its primary school closure, and students switched to online classes. Twenty minutes of online PE classes were arranged every weekday. A television channel also broadcasted two PE training classes (11:45–12:00 and 15:35–15:50) every weekday. Parents were strongly recommended to participate in physical training with their children during the broadcast. From March 2020 to July 2020, primary schools gradually resumed face-to-face classes. The education bureau of Guangzhou announced the “Guidelines of Physical and Health Education for Primary and Secondary Schools during COVID-19 Prevention and Control”. The guidelines set out the promotion of health and reinforcement of non-contact sports and physical training to enhance the students’ cardiovascular function and muscular strength. A rope-jump campaign was organised, in which grade 1 to 2 students were recommended 500 rope-jumps every day, grade 3 to 4 students 1000 jumps, and Grade 5 to 6 students three minutes. The second measurement timepoint was in November 2020 after the shutdown, and the third measurement was taken a year later.

The change in the physical fitness of students after COVID-19 seemed to be different between places and populations. In New York city, students who experienced school shutdowns had weakened cardiovascular fitness, including significant reductions in maximum oxygen uptake and oxygen uptake at anaerobic threshold [32]. Another study conducted in the United States reported a decline in push-up and sit-up performances after COVID-19, and an elevation in the BMI of both male and female eighth-grade students [33]. In Croatia, sit-up and 600 m running performance deteriorated in students after lockdown [34]. Restriction measures in Spain led to an increase in BMI, waist circumference, waist-to-hip and waist-to-height ratios along with depreciating muscular fitness in children and adolescent girls [35]. The negative findings for muscle fitness were also observed in boys [34]. Tsoukos and Bogdanis [36] reported that the upper body strength and flexibility of adolescents had worsened, especially in males. This divergence could be due to the differences in policy, culture, obedience, and governance among cities/countries [37,38,39]. 

Nevertheless, some studies demonstrated that physical fitness could be maintained via home-based individual physical training, despite increases in body fat [40]. In China, a study conducted in Fujian Province found that the aerobic fitness of students deteriorated after lockdown but vital capacity, flexibility, and muscular strength improved [41]. The authors suspected that the peculiar finding was confounded by physical growth with age [41], which was confirmed in our study, where age, gender, and BMI were significant confounding factors. 

The decline in physical flexibility reported in our study could be attributed to prolonged sedentary behaviour or screen-time among students at home during COVID-19 [42]. Prolonged mobile phone use or gaming could also lead to poor spine posture, and back and shoulder pain [43,44]. On the other hand, most investigations reported negative findings for different aspects of physical fitness [33,34,35]. However, our study found some positive outcomes, specifically lung capacity, and rope-jumping and sit-up performance, which could be easily administered at home. Sprint and shuttle run performance deteriorated since they require large outdoor spaces. Our findings for lung capacity aligned with another study conducted in China, and we believe that the online PE classes and the reinforcement measures after school resumption contributed to these positive outcomes. Health promotion programmes and policies have played important roles in the retention and enhancement of physical fitness during and after the restrictions of COVID-19 [45,46]. Dwyer, et al. [47] conducted a brief review of the policies and actions to promote physical activities in different counties and confirmed that health promotion programmes can improve health as indicated by various physical fitness variables. 

Home-based fitness could be another solution to maintain physical fitness and minimize the negative effects of sedentary behaviour (such as prolonged sitting) without specific space and equipment [48]. Besides yoga, Pilates and Tai Chi, higher intensity workouts can be entertained at home by lifting buckets, chair squats, and sit-ups [7,29]. Furthermore, high-intensity interval training (HIITT) can facilitate a higher level workout for the muscles and the cardiovascular system at home [49]. In addition, interactive home fitness apps and exergames can enhance the motivation for physical exercise, especially for students [12,48,50].

There were some limitations in this study. The research was only conducted in one primary school in a Chinese city, which may not have sufficient external validity for the province or the country. Moreover, the measurement time interval was one year and considered less susceptible to the rapid changes of the virus outbreak and policy updates. Some confounding factors, such as physical injuries, infection by COVID-19 (long COVID-19 symptoms) [51], and non-obedience due to pandemic fatigue [52] experienced between the intervals, were not taken into account. Although schools resumed around May to July, social distancing measures, including mask mandates, and closure of sports facilities, did not totally relax by that time and the policy varied in different regions of the city. These measures may have affected the students’ contemplation and participation in outdoor physical exercises or to even leave their homes. This might be supported by the fact that the trend for all outcome measures did not fully recover one year after school resumption. However, the spread/variation became larger (as shown in the plot distribution in Figure 1), which indicated that students may have behaved or responded differently. On the other hand, although existing studies had mixed outcomes, it would be difficult to compare between cities because of variations in the outbreak locations, social distancing measures and policies, education arrangements, and culture. In addition, student compliance in PE classes and other related training was not evaluated, and monitoring the physical activity and prolonged sitting time could be helpful for understanding the behaviour of students [53,54]. Apart from the assessment of physical fitness, other physical examinations should be incorporated into the annual assessment, such as scoliosis assessment [55,56] and spine mobility and stability [57], to ensure that bone growth is not affected by COVID-19 during growth of the students. Measurement of body surface area could also supplement mass and height information for a better indication of the physiological condition of the students [58]. On the other hand, the physical fitness assessment is a nationwide exercise. The big data constructed by the government could be used to develop a global national testing protocol and a normalised value/range for physical fitness for the country could inform public health measures during adverse events, such as COVID-19.

In 2022, Omicron, a highly contagious COVID-19 variant, continues to rage and ravage the world. Hong Kong and major cities in Mainland China are imposing more stringent and prolonged social distancing measures [59,60]. We recommend incorporating flexibility training exercises and interventions to reduce sitting time in online education to further strengthen the physical flexibility of students.

## 5. Conclusions

After the coronavirus (COVID-19) outbreak, our study suggested that primary school students had:

− A slight but significant elevation in body mass index;
− Poorer physical flexibility;
− Poorer sprint and shuttle run performance;
− Slight increase in lung vital capacity;
− Significant improvement in rope-jumping and sit-ups.

## Figures and Tables

**Table 1:** Assessment tasks, indicators and weights of the National Student Physical Fitness Standard of China (version 2014).

Grader	No. of Assessment	Task/Indicator	Metric	^#^ Weight (%)
All Grades	-	BMI	kg/m^2^	15
		Lung Vital Capacity	mL	15
Grade 1–2	5	50 m sprint	Time (seconds)	20
		Sit & Reach	Max Reach distance (cm)	30
		1 min rope-jumping	No. of jumps	20
Grade 3–4	6	50 m sprint	Time (seconds)	20
		Sit & Reach	Max Reach distance (cm)	20
		1 min rope-jumping	No. of jumps	20
		1 min sit-ups	No. of reps	10
Grade 5–6	7	50 m sprint	Time (seconds)	20
		Sit & Reach	Max Reach distance (cm)	10
		1 min rope-jumping	No. of jumps	10
		1 min sit-ups	No. of reps	20
		50 m × 8 shuttle run	Time (seconds)	10

^#^ These weights were used to calculate the overall physical fitness score after the normalization of each indicator according to Table S1. BMI, body mass index; No., number.

**Table 2:** Basic demographic information for the participants in three measurement timepoints (year of assessment).

	Year of Assessment		
	2019	2020	2021
Total	1916	2110	2345
Grade 1	424 **	379 *	398
Grade 2	527 **	426 **	387 *
Grade 3	425 **	530 **	434 **
Grade 4	154 **	419 **	534 **
Grade 5	202 *	163 **	429 **
Grade 6	184	193 *	163 **
Female:Male	867:1049	955:1155	1063:1282
Age	8.70 (1.66)	8.91 (1.28)	9.09 (1.59)
Height (cm)	132 (10.8)	133 (11.8)	135 (12.6)
Body mass (kg)	28.5 (8.79)	30.6 (9.76)	30.7 (8.68)
BMI (kg/m^2^)	16.1 (2.72)	16.9 (3.05)	16.6 (2.90)

Data are presented as mean (standard deviation); BMI, body mass index. * Cohort of students that had two measurements in the study; ** cohort of students that taken three measurements in the study. The number of students in each cohort may not be consistent from year to year due to exemptions, missing data, and new students transferring from another school.

**Table 3:** Effect estimates on the outcome measures by year and covariates, including gender, age, and BMI using GEE.

Outcome	Predictor	Effect (β)	95% Wald CI	*p*-Value
Overall physical fitness score	Gender = Male	−1.34	−1.83 to −0.84	<0.001
	Gender = Female (Ref)	-	-	-
	Age	1.33	0.72 to 1.95	<0.001
	BMI	−0.28	−0.37 to −0.18	<0.001
	Grader	−0.87	−1.52 to −0.22	<0.001
	Year = 2021	4.12	3.60 to 4.64	<0.001
	Year = 2020	2.12	1.73 to 2.51	<0.001
	Year = 2019 (Ref)	-	-	-
BMI	Gender = Male	0.73	0.58 to 0.88	<0.001
	Gender = Female (Ref.)	-	-	-
	Age	0.56	0.52 to 0.61	<0.001
	Year = 2021	0.39	0.24 to 0.54	<0.001
	Year = 2020	0.64	0.52 to 0.77	<0.001
	Year = 2019 (Ref)	-	-	-
Lung vital capacity	Gender = Male	97.28	74.56 to 119.96	<0.001
	Gender = Female (Ref)	-	-	-
	Age	213.06	205.45 to 220.67	<0.001
	BMI	21.15	16.24 to 26.06	<0.001
	Year = 2021	239.58	214.95 to 264.20	<0.001
	Year = 2020	153.28	130.05 to 176.52	<0.001
	Year = 2019 (Ref)	-	-	-
50 m sprint time (seconds)	Gender = Male	−0.28	−0.34 to −0.23	<0.001
	Gender = Female (Ref)	-	-	-
	Age	−0.48	−0.50 to −0.47	<0.001
	BMI	0.017	0.007 to 0.027	0.001
	Year = 2021	0.10	0.04 to 0.16	0.001
	Year = 2020	0.02	−0.03 to 0.07	0.447
	Year = 2019 (Ref)	-	-	-
Sit and reach distance (cm)	Gender = Male	−4.64	−4.97 to −4.32	<0.001
	Gender = Female (Ref)	-	-	-
	Age	0.32	0.22 to 0.43	<0.001
	BMI	0.09	0.04 to 0.15	0.001
	Year = 2021	−1.32	−1.66 to −0.98	<0.001
	Year = 2020	−0.64	−0.94 to −0.35	<0.001
	Year = 2019 (Ref)	-	-	-
Rope-jumping (counts) ^#^	Gender = Male	0.97	0.95 to 0.99	0.002
	Gender = Female (Ref)	-	-	-
	Age	1.11	1.108 to 1.120	<0.001
	BMI	1.00	0.995 to 1.001	0.181
	Year = 2021	1.39	1.38 to 1.42	<0.001
	Year = 2020	1.23	1.21 to 1.25	<0.001
	Year = 2019 (Ref)	-	-	-
Sit-up (counts) ^#^	Gender = Male	1.03	1.01 to 1.06	0.001
	Gender = Female (Ref)	-	-	-
	Age	1.08	1.07 to 1.08	<0.001
	BMI	1.00	0.998 to 1.005	0.311
	Year = 2021	1.07	1.05 to 1.10	<0.001
	Year = 2020	1.02	1.00 to 1.04	0.054
	Year = 2019 (Ref)	-	-	-
Shuttle run time (seconds)	Gender = Male	−2.98	−5.39 to −0.57	0.016
	Gender = Female (Ref)			
	Age	−1.23	−3.02 to 0.58	0.184
	BMI	0.87	0.39 to 1.35	<0.001
	Year = 2021	5.33	2.29 to 8.37	0.001
	Year = 2020	2.36	−0.23 to 4.94	0.074
	Year = 2019 (Ref)	-	-	-

^#^ The GEE model was based on a negative binomial distribution with a log link for rope-jumping and sit-up outcomes. The effects are presented as exponential estimates. BMI, body mass index.

## Data Availability

Restrictions apply to the availability of these data because the data were obtained from a third party. It could be available from the first authors (W.H. and D.L.) with the permission of the third party.

## Supplementary Materials

The following supporting information can be downloaded at: https://www.mdpi.com/article/10.3390/ijerph19137870/s1, Table S1: National Student Physical Fitness Standard of China Conversion table of indicators for the calculation of overall physical fitness score.

## References

[1] World Health Organization WHO Coronavirus (COVID-19) Dashboard. https://covid19.who.int/.

[2] Jena P.R., Majhi R., Kalli R., Managi S., Majhi B. (2021). Impact of COVID-19 on GDP of major economies: Application of the artificial neural network forecaster. Econ. Anal. Policy.

[3] Wang F., Wang J.-D. (2022). Estimating US Earnings Loss Associated with COVID-19 Based on Human Capital Calculation. Int. J. Environ. Res. Public Health.

[4] Giansanti D. (2021). The Role of the mHealth in the Fight against the COVID-19: Successes and Failures. Healthcare.

[5] Chinazzi M., Davis J.T., Ajelli M., Gioannini C., Litvinova M., Merler S., Pastore y Piontti A., Mu K., Rossi L., Sun K. (2020). The effect of travel restrictions on the spread of the 2019 novel coronavirus (COVID-19) outbreak. Science.

[6] Devi S. (2020). Travel restrictions hampering COVID-19 response. Lancet.

[7] Kaur H., Singh T., Arya Y.K., Mittal S. (2020). Physical fitness and exercise during the COVID-19 pandemic: A qualitative enquiry. Front. Psychol..

[8] Dun Y., Ripley-Gonzalez J.W., Zhou N., Li Q., Chen M., Hu Z., Zhang W., Thomas R.J., Olson T.P., Liu J. (2021). The association between prior physical fitness and depression in young adults during the COVID-19 pandemic—A cross-sectional, retrospective study. PeerJ.

[9] Adjodah D., Dinakar K., Chinazzi M., Fraiberger S.P., Pentland A., Bates S., Staller K., Vespignani A., Bhatt D.L. (2021). Association between COVID-19 outcomes and mask mandates, adherence, and attitudes. PLoS ONE.

[10] Ammar A., Brach M., Trabelsi K., Chtourou H., Boukhris O., Masmoudi L., Bouaziz B., Bentlage E., How D., Ahmed M. (2020). Effects of COVID-19 home confinement on eating behaviour and physical activity: Results of the ECLB-COVID19 international online survey. Nutrients.

[11] Makizako H., Akaida S., Shono S., Shiiba R., Taniguchi Y., Shiratsuchi D., Nakai Y. (2021). Physical activity and perceived physical fitness during the COVID-19 epidemic: A population of 40-to 69-year-olds in Japan. Int. J. Environ. Res. Public Health.

[12] Yang Y., Koenigstorfer J. (2020). Determinants of physical activity maintenance during the COVID-19 pandemic: A focus on fitness apps. Transl. Behav. Med..

[13] Szymkowiak A., Melović B., Dabić M., Jeganathan K., Kundi G.S. (2021). Information technology and Gen Z: The role of teachers, the internet, and technology in the education of young people. Technol. Soc..

[14] Lemay D.J., Bazelais P., Doleck T. (2021). Transition to online learning during the COVID-19 pandemic. Comput. Hum. Behav. Rep..

[15] Varea V., González-Calvo G. (2021). Touchless classes and absent bodies: Teaching physical education in times of COVID-19. Sport Educ. Soc..

[16] Bajramovic I., Redzepagic S., Bjelica D., Krivokapic D., Jeleskovic E., Likic S. (2020). Level of active lifestyle and exercise approach among sports-active female students of the University of Sarajevo during the COVID-19 pandemic. J. Anthropol. Sport Phys. Educ..

[17] Tornaghi M., Lovecchio N., Vandoni M., Chirico A., Codella R. (2020). Physical activity levels across COVID-19 outbreak in youngsters of Northwestern Lombardy. J. Sports Med. Phys. Fit..

[18] Stockwell S., Trott M., Tully M., Shin J., Barnett Y., Butler L., McDermott D., Schuch F., Smith L. (2021). Changes in physical activity and sedentary behaviours from before to during the COVID-19 pandemic lockdown: A systematic review. BMJ Open Sport Exerc. Med..

[19] Margaritis I., Houdart S., El Ouadrhiri Y., Bigard X., Vuillemin A., Duché P. (2020). How to deal with COVID-19 epidemic-related lockdown physical inactivity and sedentary increase in youth? Adaptation of Anses’ benchmarks. Arch. Public Health.

[20] Faigenbaum A.D., Farrell A.C., Fabiano M., Radler T.A., Naclerio F., Ratamess N.A., Kang J., Myer G.D. (2013). Effects of detraining on fitness performance in 7-year-old children. J. Strength Cond. Res..

[21] McCall B. (2020). Shut down and reboot—Preparing to minimise infection in a post-COVID-19 era. Lancet Digit. Health.

[22] Ministry of Education of the People’s Republic of China Notice of the Ministry of Education on the National Student Physical Fitness Standard (Revised 2014). http://www.moe.gov.cn/s78/A17/twys_left/moe_938/moe_792/s3273/201407/t20140708_171692.html.

[23] Nystad W., Samuelsen S., Nafstad P., Edvardsen E., Stensrud T., Jaakkola J. (2002). Feasibility of measuring lung function in preschool children. Thorax.

[24] Song P., Li X., Gasevic D., Flores A.B., Yu Z. (2016). BMI, waist circumference reference values for Chinese school-aged children and adolescents. Int. J. Environ. Res. Public Health.

[25] Lee I.-M., Shiroma E.J., Lobelo F., Puska P., Blair S.N., Katzmarzyk P.T., Group L.P.A.S.W. (2012). Effect of physical inactivity on major non-communicable diseases worldwide: An analysis of burden of disease and life expectancy. Lancet.

[26] Jurak G., Morrison S.A., Leskošek B., Kovač M., Hadžić V., Vodičar J., Truden P., Starc G. (2020). Physical activity recommendations during the coronavirus disease-2019 virus outbreak. J. Sport Health Sci..

[27] Jarvis C.I., Van Zandvoort K., Gimma A., Prem K., Klepac P., Rubin G.J., Edmunds W.J. (2020). Quantifying the impact of physical distance measures on the transmission of COVID-19 in the UK. BMC Med..

[28] Jiménez-Pavón D., Carbonell-Baeza A., Lavie C.J. (2020). Physical exercise as therapy to fight against the mental and physical consequences of COVID-19 quarantine: Special focus in older people. Prog. Cardiovasc. Dis..

[29] Woods J.A., Hutchinson N.T., Powers S.K., Roberts W.O., Gomez-Cabrera M.C., Radak Z., Berkes I., Boros A., Boldogh I., Leeuwenburgh C. (2020). The COVID-19 pandemic and physical activity. Sports Med. Health Sci..

[30] Yan Z., Spaulding H.R. (2020). Extracellular superoxide dismutase, a molecular transducer of health benefits of exercise. Redox Biol..

[31] Filgueira T.O., Castoldi A., Santos L.E.R., de Amorim G.J., de Sousa Fernandes M.S., Anastácio W.D.L.D.N., Campos E.Z., Santos T.M., Souto F.O. (2021). The relevance of a physical active lifestyle and physical fitness on immune defense: Mitigating disease burden, with focus on COVID-19 consequences. Front. Immunol..

[32] Dayton J.D., Ford K., Carroll S.J., Flynn P.A., Kourtidou S., Holzer R.J. (2021). The deconditioning effect of the COVID-19 pandemic on unaffected healthy children. Pediatric Cardiol..

[33] Wahl-Alexander Z., Camic C.L. (2021). Impact of COVID-19 on school-aged male and female health-related fitness markers. Pediatric Exerc. Sci..

[34] Sunda M., Gilic B., Peric I., Jurcev Savicevic A., Sekulic D. (2021). Evidencing the influence of the COVID-19 pandemic and imposed lockdown measures on fitness status in adolescents: A preliminary report. Healthcare.

[35] Rúa-Alonso M., Rial-Vázquez J., Nine I., Lete-Lasa J.R., Clavel I., Giráldez-García M.A., Rodríguez-Corral M., Dopico-Calvo X., Iglesias-Soler E. (2022). Comparison of Physical Fitness Profiles Obtained before and during COVID-19 Pandemic in Two Independent Large Samples of Children and Adolescents: DAFIS Project. Int. J. Environ. Res. Public Health.

[36] Tsoukos A., Bogdanis G.C. (2021). The effects of a five-month lockdown due to COVID-19 on physical fitness parameters in adolescent students: A comparison between cohorts. Int. J. Environ. Res. Public Health.

[37] Agusi E.R., Ijoma S.I., Nnochin C.S., Njoku-Achu N.O., Nwosuh C.I., Meseko C.A. (2020). The COVID-19 pandemic and social distancing in Nigeria: Ignorance or defiance. Pan Afr. Med. J..

[38] Vo H.-L., Nguyen H.A.S., Nguyen K.N., Nguyen H.L.T., Nguyen H.T., Nguyen L.H., Vu G.T., Le H.T. (2020). Adherence to social distancing measures for controlling COVID-19 pandemic: Successful lesson from Vietnam. Front. Public Health.

[39] Gao J., Zhang P. (2021). China’s Public Health Policies in Response to COVID-19: From an “Authoritarian” Perspective. Front. Public Health.

[40] Parpa K., Michaelides M. (2021). The impact of COVID-19 lockdown on professional soccer players’ body composition and physical fitness. Biol. Sport.

[41] Zhou T., Zhai X., Wu N., Koriyama S., Wang D., Jin Y., Li W., Sawada S.S., Fan X. (2022). Changes in Physical Fitness during COVID-19 Pandemic Lockdown among Adolescents: A Longitudinal Study. Healthcare.

[42] Son S.-M. (2021). Correlation Study among the Bedtime Procrastination and Sleep Disorder, and Depression of University Students during COVID-19 Pandemic in Korea. Ann. Rom. Soc. Cell Biol..

[43] Lam W.-K., Liu R.-T., Chen B., Huang X.-Z., Yi J., Wong D.W.-C. (2022). Health Risks and Musculoskeletal Problems of Elite Mobile Esports Players: A Cross-Sectional Descriptive Study. Sports Med.-Open.

[44] Mustafaoglu R., Yasaci Z., Zirek E., Griffiths M.D., Ozdincler A.R. (2021). The relationship between smartphone addiction and musculoskeletal pain prevalence among young population: A cross-sectional study. Korean J. Pain.

[45] Elnaggar R.K., Alqahtani B.A., Mahmoud W.S., Elfakharany M.S. (2020). Physical activity in adolescents during the social distancing policies of the COVID-19 pandemic. Asia Pac. J. Public Health.

[46] Li M.H., Rudd J., Chow J.Y., Sit C.H.P., Wong S.H.S., Sum R.K.W. (2022). A Randomized Controlled Trial of a Blended Physical Literacy Intervention to Support Physical Activity and Health of Primary School Children. Sports Med.-Open.

[47] Dwyer M.J., Pasini M., De Dominicis S., Righi E. (2020). Physical activity: Benefits and challenges during the COVID-19 pandemic. Scand. J. Med. Sci. Sports.

[48] Nyenhuis S.M., Greiwe J., Zeiger J.S., Nanda A., Cooke A. (2020). Exercise and fitness in the age of social distancing during the COVID-19 pandemic. J. Allergy Clin. Immunol. Pract..

[49] Schwendinger F., Pocecco E. (2020). Counteracting physical inactivity during the COVID-19 pandemic: Evidence-based recommendations for home-based exercise. Int. J. Environ. Res. Public Health.

[50] Rasheed A., Abduljawad R., Mabrouk S., Jdaitawi M., Abdulmonem M. (2021). Physical fitness training program using electronic simulation games to foster psychological health among university students during COVID-19 pandemic. Int. J. Hum. Mov. Sports Sci..

[51] López-Bueno R., Calatayud J., Andersen L.L., Casaña J., Ezzatvar Y., Casajús J.A., López-Sánchez G.F., Smith L. (2021). Cardiorespiratory fitness in adolescents before and after the COVID-19 confinement: A prospective cohort study. Eur. J. Pediatrics.

[52] Petherick A., Goldszmidt R., Andrade E.B., Furst R., Hale T., Pott A., Wood A. (2021). A worldwide assessment of changes in adherence to COVID-19 protective behaviours and hypothesized pandemic fatigue. Nat. Hum. Behav..

[53] Cheung C.-W.J., Chan W.-H.R., Chiu M.-W., Law S.-Y., Lee T.-H., Zheng Y.-P. A three-month study of fall and physical activity levels of intellectual disability using a transfer belt-based motion recording sensor. Proceedings of the 6th World Congress of Biomechanics (WCB 2010).

[54] Chastin S., Culhane B., Dall P. (2014). Comparison of self-reported measure of sitting time (IPAQ) with objective measurement (activPAL). Physiol. Meas..

[55] Lee T.T.-Y., Cheung J.C.-W., Law S.-Y., To M.K.T., Cheung J.P.Y., Zheng Y.-P. (2020). Analysis of sagittal profile of spine using 3D ultrasound imaging: A phantom study and preliminary subject test. Comput. Methods Biomech. Biomed. Eng. Imaging Vis..

[56] Zheng Y., Cheung J.C.W. (2014). Three-Dimensional (3D) Ultrasound Imaging System for Assessing Scoliosis. U.S. Patent.

[57] Lam W.-K., Chen B., Liu R.-T., Cheung J.C.-W., Wong D.W.-C. (2022). Spine Posture, Mobility, and Stability of Top Mobile Esports Athletes: A Case Series. Biology.

[58] Smith L.S. (2019). Take a deeper look into body surface area. Nursing.

[59] Luo M., Liu Q., Wang J., Gong Z. (2021). From SARS to the Omicron variant of COVID-19: China’s policy adjustments and changes to prevent and control infectious diseases. BioScience Trends.

[60] Ma A., Parry J. (2022). When Hong Kong’s “dynamic zero” COVID-19 strategy met omicron, low vaccination rates sent deaths soaring. BMJ.

