# Supplementary material for: Physical Fitness of Chinese Primary School Students across the Coronavirus (COVID-19) Outbreak: A Retrospective Repeated Cross-Sectional Study
Source: Int J Environ Res Public Health. 2022 Jun 27;19(13):7870. doi: 10.3390/ijerph19137870 (PMC9265837; doi:10.3390/ijerph19137870)
Supplement: Supplementary file 1 [file ijerph-19-07870-s001.zip › ijerph-1731283-supplementary.pdf]

Table S1. National Student Physical Fitness Standard of China Conversion table of indicators for the calculation of overall physical fitness score.

Body Mass Index (Male)

Normalized Score	Grader 1	2	3	4	5	6
100	13.5~18.1	13.7~18.4	13.9~19.4	14.2~20.1	14.4~21.4	14.7~21.8
80	≤13.4	≤13.6	≤13.8	≤14.1	≤14.3	≤14.6
80	18.2~20.3	18.5~20.4	19.5~22.1	20.2~22.6	21.5~24.1	21.9~24.5
60	≥20.4	≥20.5	≥22.2	≥22.7	≥24.2	≥24.6

Body Mass Index (Female)

Normalized Score	Grader 1	2	3	4	5	6
100	13.3~17.3	13.5~17.8	13.6~18.6	13.7~19.4	13.8~20.5	14.2~20.8
80	≤13.2	≤13.4	≤13.5	≤13.6	≤13.7	≤14.1
80	17.4~19.2	17.9~20.2	18.7~21.1	19.5~22.0	20.6~22.9	20.9~23.6
60	≥19.3	≥20.3	≥21.2	≥22.1	≥23.0	≥23.7

Lung Vital Capacity (Male)

Normalized Score	Grader 1	2	3	4	5	6
100	1700	2000	2300	2600	2900	3200
95	1600	1900	2200	2500	2800	3100
90	1500	1800	2100	2400	2700	3000
85	1400	1650	1900	2150	2450	2750
80	1300	1500	1700	1900	2200	2500
78	1240	1430	1620	1820	2110	2400
76	1180	1360	1540	1740	2020	2300
74	1120	1290	1460	1660	1930	2200
72	1060	1220	1380	1580	1840	2100
70	1000	1150	1300	1500	1750	2000
68	940	1080	1220	1420	1660	1900
66	880	1010	1140	1340	1570	1800
64	820	940	1060	1260	1480	1700
62	760	870	980	1180	1390	1600
60	700	800	900	1100	1300	1500
50	660	750	840	1030	1220	1410
40	620	700	780	960	1140	1320
30	580	650	720	890	1060	1230
20	540	600	660	820	980	1140
10	500	550	600	750	900	1050

Lung Vital Capacity (Female)

Normalized Score	Grader					
	1	2	3	4	5	6
100	1400	1600	1800	2000	2250	2500
95	1300	1500	1700	1900	2150	2400
90	1200	1400	1600	1800	2050	2300
85	1100	1300	1500	1700	1950	2200
80	1000	1200	1400	1600	1850	2100
78	960	1150	1340	1530	1770	2010
76	920	1100	1280	1460	1690	1920
74	880	1050	1220	1390	1610	1830
72	840	1000	1160	1320	1530	1740
70	800	950	1100	1250	1450	1650
68	760	900	1040	1180	1370	1560
66	720	850	980	1110	1290	1470
64	680	800	920	1040	1210	1380
62	640	750	860	970	1130	1290
60	600	700	800	900	1050	1200
50	580	680	780	880	1020	1170
40	560	660	760	860	990	1140
30	540	640	740	840	960	1110
20	520	620	720	820	930	1080
10	500	600	700	800	900	1050

50m Sprint (Male)

Normalized Score	Grader					
	1	2	3	4	5	6
100	10.2	9.6	9.1	8.7	8.4	8.2
95	10.3	9.7	9.2	8.8	8.5	8.3
90	10.4	9.8	9.3	8.9	8.6	8.4
85	10.5	9.9	9.4	9	8.7	8.5
80	10.6	10	9.5	9.1	8.8	8.6
78	10.8	10.2	9.7	9.3	9	8.8
76	11	10.4	9.9	9.5	9.2	9
74	11.2	10.6	10.1	9.7	9.4	9.2
72	11.4	10.8	10.3	9.9	9.6	9.4
70	11.6	11	10.5	10.1	9.8	9.6
68	11.8	11.2	10.7	10.3	10	9.8
66	12	11.4	10.9	10.5	10.2	10
64	12.2	11.6	11.1	10.7	10.4	10.2
62	12.4	11.8	11.3	10.9	10.6	10.4
60	12.6	12	11.5	11.1	10.8	10.6
50	12.8	12.2	11.7	11.3	11	10.8
40	13	12.4	11.9	11.5	11.2	11
30	13.2	12.6	12.1	11.7	11.4	11.2

20	13.4	12.8	12.3	11.9	11.6	11.4
10	13.6	13	12.5	12.1	11.8	11.6

50m Sprint (Female)

Normalized	Grader					
Score	1	2	3	4	5	6
100	11	10	9.2	8.7	8.3	8.2
95	11.1	10.1	9.3	8.8	8.4	8.3
90	11.2	10.2	9.4	8.9	8.5	8.4
85	11.5	10.5	9.7	9.2	8.8	8.7
80	11.8	10.8	10	9.5	9.1	9
78	12	11	10.2	9.7	9.3	9.2
76	12.2	11.2	10.4	9.9	9.5	9.4
74	12.4	11.4	10.6	10.1	9.7	9.6
72	12.6	11.6	10.8	10.3	9.9	9.8
70	12.8	11.8	11	10.5	10.1	10
68	13	12	11.2	10.7	10.3	10.2
66	13.2	12.2	11.4	10.9	10.5	10.4
64	13.4	12.4	11.6	11.1	10.7	10.6
62	13.6	12.6	11.8	11.3	10.9	10.8
60	13.8	12.8	12	11.5	11.1	11
50	14	13	12.2	11.7	11.3	11.2
40	14.2	13.2	12.4	11.9	11.5	11.4
30	14.4	13.4	12.6	12.1	11.7	11.6
20	14.6	13.6	12.8	12.3	11.9	11.8
10	14.8	13.8	13	12.5	12.1	12

Sit-and-Reach (Male)

Normalized	Grader					
Score	1	2	3	4	5	6
100	16.1	16.2	16.3	16.4	16.5	16.6
95	14.6	14.7	14.9	15	15.2	15.3
90	13	13.2	13.4	13.6	13.8	14
85	12	11.9	11.8	11.7	11.6	11.5
80	11	10.6	10.2	9.8	9.4	9
78	9.9	9.5	9.1	8.6	8.2	7.7
76	8.8	8.4	8	7.4	7	6.4
74	7.7	7.3	6.9	6.2	5.8	5.1
72	6.6	6.2	5.8	5	4.6	3.8
70	5.5	5.1	4.7	3.8	3.4	2.5
68	4.4	4	3.6	2.6	2.2	1.2
66	3.3	2.9	2.5	1.4	1	-0.1
64	2.2	1.8	1.4	0.2	-0.2	-1.4
62	1.1	0.7	0.3	-1	-1.4	-2.7

60	0	-0.4	-0.8	-2.2	-2.6	-4
50	-0.8	-1.2	-1.6	-3.2	-3.6	-5
40	-1.6	-2	-2.4	-4.2	-4.6	-6
30	-2.4	-2.8	-3.2	-5.2	-5.6	-7
20	-3.2	-3.6	-4	-6.2	-6.6	-8
10	-4	-4.4	-4.8	-7.2	-7.6	-9

Sit-and-Reach (Female)

**Normalized**			**Grader**			
**Score**	**1**	**2**	**3**	**4**	**5**	**6**
100	18.6	18.9	19.2	19.5	19.8	19.9
95	17.3	17.6	17.9	18.1	18.5	18.7
90	16	16.3	16.6	16.9	17.2	17.5
85	14.7	14.8	14.9	15	15.1	15.2
80	13.4	13.3	13.2	13.1	13	12.9
78	12.3	12.2	12.1	12	11.9	11.8
76	11.2	11.1	11	10.9	10.8	10.7
74	10.1	10	9.9	9.8	9.7	9.6
72	9	8.9	8.8	8.7	8.6	8.5
70	7.9	7.8	7.7	7.6	7.5	7.4
68	6.8	6.7	6.6	6.5	6.4	6.3
66	5.7	5.6	5.5	5.4	5.3	5.2
64	4.6	4.5	4.4	4.3	4.2	4.1
62	3.5	3.4	3.3	3.2	3.1	3
60	2.4	2.3	2.2	2.1	2	1.9
50	1.6	1.5	1.4	1.3	1.2	1.1
40	0.8	0.7	0.6	0.5	0.4	0.3
30	0	-0.1	-0.2	-0.3	-0.4	-0.5
20	-0.8	-0.9	-1	-1.1	-1.2	-1.3
10	-1.6	-1.7	-1.8	-1.9	-2	-2.1

Rope-jumping (Male)

**Normalized**			**Grader**			
**Score**	**1**	**2**	**3**	**4**	**5**	**6**
100	109	117	126	137	148	157
95	104	112	121	132	143	152
90	99	107	116	127	138	147
85	93	101	110	121	132	141
80	87	95	104	115	126	135
78	80	88	97	108	119	128
76	73	81	90	101	112	121
74	66	74	83	94	105	114
72	59	67	76	87	98	107
70	52	60	69	80	91	100

68	45	53	62	73	84	93
66	38	46	55	66	77	86
64	31	39	48	59	70	79
62	24	32	41	52	63	72
60	17	25	34	45	56	65
50	14	22	31	42	53	62
40	11	19	28	39	50	59
30	8	16	25	36	47	56
20	5	13	22	33	44	53
10	2	10	19	30	41	50

Rope-jumping (Female)

**Normalized Score**	**Grader**					
	**1**	**2**	**3**	**4**	**5**	**6**
100	117	127	139	149	158	166
95	110	120	132	142	151	159
90	103	113	125	135	144	152
85	95	105	117	127	136	144
80	87	97	109	119	128	136
78	80	90	102	112	121	129
76	73	83	95	105	114	122
74	66	76	88	98	107	115
72	59	69	81	91	100	108
70	52	62	74	84	93	101
68	45	55	67	77	86	94
66	38	48	60	70	79	87
64	31	41	53	63	72	80
62	24	34	46	56	65	73
60	17	27	39	49	58	66
50	14	24	36	46	55	63
40	11	21	33	43	52	60
30	8	18	30	40	49	57
20	5	15	27	37	46	54
10	2	12	24	34	43	51

Rope-jumping Extra Bonus after exceeding 100 in rope-jumping item (Male)

**Normalized Score**	**Grader**					
	**1**	**2**	**3**	**4**	**5**	**6**
20	40	40	40	40	40	40
19	38	38	38	38	38	38
18	36	36	36	36	36	36
17	34	34	34	34	34	34
16	32	32	32	32	32	32
15	30	30	30	30	30	30

14	28	28	28	28	28	28
13	26	26	26	26	26	26
12	24	24	24	24	24	24
11	22	22	22	22	22	22
10	20	20	20	20	20	20
9	18	18	18	18	18	18
8	16	16	16	16	16	16
7	14	14	14	14	14	14
6	12	12	12	12	12	12
5	10	10	10	10	10	10
4	8	8	8	8	8	8
3	6	6	6	6	6	6
2	4	4	4	4	4	4
1	2	2	2	2	2	2

Rope-jumping Extra Bonus after exceeding 100 in rope-jumping item (Female)

**Normalized**			**Grader**			
**Score**	**1**	**2**	**3**	**4**	**5**	**6**
20	40	40	40	40	40	40
19	38	38	38	38	38	38
18	36	36	36	36	36	36
17	34	34	34	34	34	34
16	32	32	32	32	32	32
15	30	30	30	30	30	30
14	28	28	28	28	28	28
13	26	26	26	26	26	26
12	24	24	24	24	24	24
11	22	22	22	22	22	22
10	20	20	20	20	20	20
9	18	18	18	18	18	18
8	16	16	16	16	16	16
7	14	14	14	14	14	14
6	12	12	12	12	12	12
5	10	10	10	10	10	10
4	8	8	8	8	8	8
3	6	6	6	6	6	6
2	4	4	4	4	4	4
1	2	2	2	2	2	2

Sit-ups (Male)

**Normalized**			**Grader**	
**Score**	**3**	**4**	**5**	**6**

100	48	49	50	51
95	45	46	47	48
90	42	43	44	45
85	39	40	41	42
80	36	37	38	39
78	34	35	36	37
76	32	33	34	35
74	30	31	32	33
72	28	29	30	31
70	26	27	28	29
68	24	25	26	27
66	22	23	24	25
64	20	21	22	23
62	18	19	20	21
60	16	17	18	19
50	14	15	16	17
40	12	13	14	15
30	10	11	12	13
20	8	9	10	11
10	6	7	8	9

Sit-ups (Female)

**Normalized**			**Grader**	
**Score**	**3**	**4**	**5**	**6**
100	46	47	48	49
95	44	45	46	47
90	42	43	44	45
85	39	40	41	42
80	36	37	38	39
78	34	35	36	37
76	32	33	34	35
74	30	31	32	33
72	28	29	30	31
70	26	27	28	29
68	24	25	26	27
66	22	23	24	25
64	20	21	22	23
62	18	19	20	21
60	16	17	18	19
50	14	15	16	17
40	12	13	14	15
30	10	11	12	13
20	8	9	10	11
10	6	7	8	9

Shuttle Run (Male)

Normalized Score	Grader	
	5	6
100	1'36"	1'30"
95	1'39"	1'33"
90	1'42"	1'36"
85	1'45"	1'39"
80	1'48"	1'42"
78	1'51"	1'45"
76	1'54"	1'48"
74	1'57"	1'51"
72	2'00"	1'54"
70	2'03"	1'57"
68	2'06"	2'00"
66	2'09"	2'03"
64	2'12"	2'06"
62	2'15"	2'09"
60	2'18"	2'12"
50	2'22"	2'16"
40	2'26"	2'20"
30	2'30"	2'24"
20	2'34"	2'28"
10	2'38"	2'32"

Shuttle Run (Female)

Normalized Score	Grader	
	5	6
100	1'41"	1'37"
95	1'44"	1'40"
90	1'47"	1'43"
85	1'50"	1'46"
80	1'53"	1'49"
78	1'56"	1'52"
76	1'59"	1'55"
74	2'02"	1'58"
72	2'05"	2'01"
70	2'08"	2'04"
68	2'11"	2'07"
66	2'14"	2'10"
64	2'17"	2'13"
62	2'20"	2'16"
60	2'23"	2'19"
50	2'27"	2'23"
40	2'31"	2'27"
30	2'35"	2'31"

20	2'39"	2'35"
10	2'43"	2'39"
